# **Proposal to extend the PROMIS**® **item bank v2.0 ‘Ability to Participate in Social Roles and Activities’: item generation and content validity**

**DOI:** 10.1007/s11136-020-02540-3

**Published:** 2020-06-02

**Authors:** Lisette M. van Leeuwen, Sietske J. Tamminga, Margarita Ravinskaya, Astrid de Wind, Elisabeth A. Hahn, Caroline B. Terwee, Heleen Beckerman, Edwin J. Boezeman, Jan L. Hoving, Maaike A. Huysmans, Karen Nieuwenhuijsen, Angela G. E. M. de Boer, Allard J. van der Beek

**Affiliations:** 1 Amsterdam UMC, Vrije Universiteit Amsterdam, Department of Otolaryngology-Head and Neck Surgery, Ear & Hearing, Amsterdam Public Health Research Institute, De Boelelaan, 1081 BT Amsterdam, Netherlands; 2 Amsterdam UMC, University of Amsterdam, Coronel Institute of Occupational Health, Amsterdam Public Health Research Institute, Meibergdreef, Amsterdam, Netherlands; 3 Northwestern University Feinberg School of Medicine, Department of Medical Social Sciences, Chicago, IL USA; 4 Amsterdam UMC, Vrije Universiteit Amsterdam, Department of Epidemiology and Biostatistics, Amsterdam Public Health Research Institute, De Boelelaan, Amsterdam, Netherlands; 5 Amsterdam UMC, Vrije Universiteit Amsterdam, Department of Rehabilitation Medicine, Amsterdam Public Health Research Institute, De Boelelaan, Amsterdam, Netherlands; 6 Amsterdam UMC, Vrije Universiteit Amsterdam, Department of Public and Occupational Health, Amsterdam Public Health Research Institute, De Boelelaan, Amsterdam, Netherlands

**Keywords:** Social participation, Content validity, PROMIS®

## Abstract

**Purpose:**

Previous research indicated that the Patient-Reported Outcomes Measurement Information System (PROMIS®) item bank v2.0 ‘Ability to Participate in Social Roles and Activities’ may miss subdomains of social participation. The purpose of this study was to generate items for these missing subdomains and to evaluate their content validity.

**Methods:**

A three-step approach was followed: (1) Item generation for 16 International Classification of Functioning Disability and Health subdomains currently not covered by the item bank; (2) Evaluation of content validity of generated items through expert review (*n* = 20) and think-aloud interviews with a purposeful sample of people with and without (chronic) health conditions (*n* = 10), to assess item comprehensibility, relevance, and comprehensiveness; and 3) Item revision based on the results of step 2, in a consensus procedure.

**Results:**

First, 48 items were generated. Second, overall, content experts indicated that the generated items were relevant. Furthermore, based on experts’ responses, items were simplified and ‘participation in social media’ was identified as an important additional subdomain of social participation. Additionally, ‘participating in various social roles simultaneously’ was identified as a missing item. Based on the responses of the interviewed adults items were simplified. Third, in total 17 items, covering 17 subdomains, were proposed to be added to the original item bank.

**Discussion:**

The relevance, comprehensibility and comprehensiveness of the 17 proposed items were supported. Whether the proposed extension of the item bank leads to better psychometric properties of the item bank should be tested in a large-scale field study.

**Electronic supplementary material:**

The online version of this article (10.1007/s11136-020-02540-3) contains supplementary material, which is available to authorized users.

## Introduction

Participation in social roles and activities (or social participation) is an important determinant of health [1]. Specifically, participation restrictions are negatively associated with quality of life outcomes, social inclusion, and successful aging [2–4]. In addition, higher levels of social participation have been found to protect against physical and mental illnesses and facilitate recovery from disease [5]. The concept of participation was introduced by the World Health Organization as: “an individual’s ‘involvement in life situations’, where participation is defined in relation to an individual’s health condition, body functions and structures, activities and contextual factors” (International Classification of Functioning Disability and Health (ICF) [6]). These life situations include interpersonal relationships, major life areas (e.g., employment), as well as recreation, leisure and community life [6]. With the growing number of chronic conditions and longevity, optimizing opportunities for social participation is increasingly being called for [7]. To be able to develop and evaluate interventions that can improve social participation, a measurement instrument is required that takes into account the diversity and dynamic nature of the construct social participation, applicable to individuals with and without (chronic) health conditions. However, social participation is difficult to measure as it involves a diversity of subdomains (such as work and socializing), which relevance may vary between individuals and over time [8]. The meaning of participation often seems to depend on the purpose of a given clinical or research program [9]. Furthermore, there is a lack of consensus on the definition of participation and available measures reflect the varying conceptual differences in constructs being measured [10].

The Patient-Reported Outcomes Measurement Information System (PROMIS®) item bank v2.0 ‘Ability to Participate in Social Roles and Activities’ measures social participation across health conditions and settings [11]. It was developed based on Item Response Theory (IRT) [11]. An advantage of IRT is that subsets of items can be administered as short forms or as a Computerized Adaptive Test (CAT). In a CAT, the successive items are chosen based on given answers to previous items, enabling individuals only to respond to a minimal number of relevant items, making it less burdensome to fill in. Also, in IRT-based item banks, items can be removed or added without changing the underlying metric and the adapted item bank maintains comparable with scores using older versions of the measurement instrument [12]. This is a significant advantage over measurement instruments based on classical test theory [13], where making changes can have important consequences to the interpretation of the measurement instrument. Being able to make changes while maintaining comparability with older versions of the instrument is helpful for the measurement of social participation. For example, social innovations such as internet-enabled communication can have an impact on how restrictions in interpersonal relationships are experienced [14].

An overview of the characteristics of the PROMIS® item bank ‘Ability to Participate in Social Roles and Activities’ is presented in Table 1. Validation of the item bank is ongoing and recommended through both qualitative and quantitative efforts [15]. The item bank has been applied in different settings (e.g., rheumatoid arthritis [16], heart failure [17], and abdominal surgery [18]). The item bank was also translated into Dutch-Flemish [19]. Sufficient psychometric properties of this item bank were found in the Dutch general population [20]. However, previous research into the meaning of participation [8] indicated that the current item bank does not cover all subdomains that were found important by the general Dutch population, which may hamper the content validity and measurement precision of the item bank. Items were missed within the following ICF domains: domestic life, interpersonal relationships, economic life, recreation, community life, and social and civic life [8]. Previous research supports that participation constitutes a variety of subdomains [21–23]. Previous research has also found the PROMIS® item bank did not cover all subcategories of the ICF [24]. Moreover, Tucker et al. concludes that mapping between PROMIS® and the ICF helps clarify measurement opportunities and that may lead to improved, comprehensive health outcome measures. Furthermore, the study by Terwee and colleagues found that a substantial number of adults scored the highest level of participation, indicating a ceiling effect [20]. This means that when participating at a high level, an improvement in participation cannot be measured. The quality of the item bank may therefore benefit from adding items that cover all subdomains of participation meaningful to people with an without (chronic) health conditions, as well as from adding items that are relevant for people with a high level of participation. In order to address these issues, the purpose of the present study was to generate items to be added to the existing item bank and to evaluate the content validity of the proposed extension of the item bank.

**Table 1:** Characteristics of the PROMIS® item bank ‘Ability to Participate in Social Roles and Activities’

Definition	The perceived ability to perform one’s usual social roles and activities
PROMIS domain	Social Health
PROMIS subdomain	Social Function
Developers	PROMIS® Social Health Workgroup [9, 11, 15]
Target population	Healthy people, as well as those with a range of physical and mental health conditions
Number of items	35 items covering 6 ICF subdomains (i.e., household tasks, assisting others, informal social relationships, family relationships, work and employment, and socializing) [11]
Wording	All 35 items are worded in terms of perceived restrictions, e.g., “I have trouble doing my regular daily work around the house”
Response categories	5-point Likert response scale (ranging from ‘never’ to ‘always’). Responses are reverse-coded, so that high scores represent a high level of participation
Time frame	No time frame is included in the items

## Methods

**Fig. 1:**
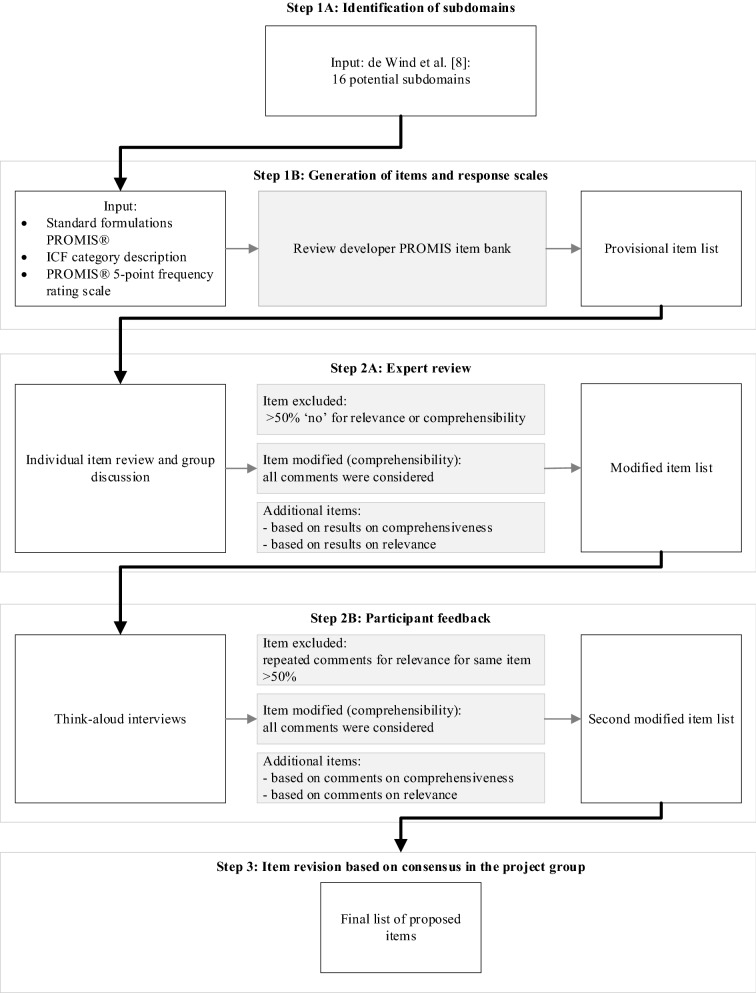
Approach of item generation and content validity evaluation

The generation and testing of the items were guided by the PROMIS® scientific standards [25, 26], presented in Fig. 1. The Medical Ethical Committee of Amsterdam UMC, location VU University Medical Center (Amsterdam, the Netherlands), declared that the Medical Research Involving Human Subjects Act does not apply to this study, and had no objection to execution of this study (reference number 2018.513).

### Step 1: item generation

#### A) Identification of subdomains

The potential subdomains to be added to the existing item bank was based on the previous qualitative interview study [8]. The set of possible subdomains generated from the interview transcripts was organized using the ICF classification. Table 2 presents the identified ICF subdomains and corresponding ICF domains.

**Table 2:** Potential subdomains identified by De Wind et al. [8] currently not covered in the item bank ‘Ability to Participate in Social Roles and Activities’

ICF domain	ICF subdomain (ICF code)
Domestic life	Acquiring a place to live (d610)
	Acquisition of goods and services (d620)
	Caring for household objects (d650)^a^
	Assisting others^a^
Interpersonal relationships	Relating with strangers (d730)
	Formal relationships (d740)
	Romantic relationships (d760)
Major life areas	Education life (d810–d839)
	Remunerative employment (d850)^a^
	Non-remunerative employment (d855)^a^
Economic life	Basic economic transactions (d860)
	Complex economic transactions (d870)
Community life, social and civic life	Community life (d910)
	Religion and spirituality (d930)
	Political life and citizenship (d950)
	Recreation and leisure (d920)

^a^In the original PROMIS® item bank v2.0 ‘Ability to Participate in Social Roles and Activities’ these subdomains are taken together in the formulation of the items. Previous work [8] shows that a distinction between these subdomains may be desirable, so separate items could be considered for each subdomain

#### B) Generation of items and response scales

Dutch items were generated for the subdomains (Table 1). The ICF category description and the participants’ quotes [8] were used to define the specific content of the item. The method to formulate items involved a formal decision-making and consensus process in the project team, consisting of researchers with relevant expertise in the field of participation research and outcome measurement. The *sentence structure* was guided by PROMIS® standards and the formulations that are included in the original item bank (stems, responses, tense and person). For example, these included “I have trouble..”/ “I have to limit..”/ “I feel limited in..”. For each subdomain, multiple items were formulated for further content validity evaluation (step 2), using all different formulations that were applicable to the subdomains to be measured. The identified subdomains and the formulated items were discussed with the initial developer of the item bank (EH), and the items were modified based on her feedback. Finally, step 1B resulted in a provisional item list agreed upon within the project team.

The response choices were adopted form the standard PROMIS® 5-point frequency rating scale: ‘never’, ‘rarely’, ‘sometimes’, ‘usually’, ‘always’.

### Step 2: content validity

The aim of this second step was to test whether the items in the provisional list were relevant (i.e., all items should be relevant for the construct of interest), comprehensive (i.e., no key aspects of the construct should be missing), and comprehensible (i.e., the items should be understood by the target population as intended) [27]. The provisional item list was discussed in a group of relevant stakeholders within societal participation and health-related research (i.e., “content experts”) and tested in a purposeful sample of people with and without (chronic) health conditions.

#### A) Review by content experts

To discuss the provisional item list with content experts, we organized a session with members of the research program “Societal Participation and Health” of the Amsterdam Public Health research institute. The session consisted of four successive parts, presented in Table 3.

**Table 3:** Session expert review

Parts	Content/goals
1. Plenary presentation	Background information on PROMIS® and the development of item banks
2. Plenary presentation	Previous results (i.e., based on De Wind et al. [8]), current status of the project, and the goals of the group discussion
3. Individual assignment	The experts were provided with the provisional item list and the original item bank, and were asked to rate each provisional item for its relevance (yes/no) and comprehensibility (yes/no) The experts were placed in small groups (i.e., 4–5 people) and encouraged to discuss their results with each other
4. Plenary group discussion	Main results were discussed to identify: a. common problems with the formulation of the items (i.e., comprehensibility); b. subdomains of participation not yet covered in the provisional item list (i.e., comprehensiveness) or original item bank; c. additional items that may be relevant for individuals with high levels of social participation (i.e., relevance); d. discussion on subdomain scores (i.e., relevance)

For analysis of the rating of the items by the experts (part 3), a pre-set decision rule of < 50% relevance and comprehensiveness score was used to indicate if items should be removed or changed (guided by the European Organization for Research and Treatment of Cancer (EORTC) Quality of Life Group) [28]. For analysis of the discussion, qualitative content analysis was used to group the comments around key issues [29]. This content analysis further guided decisions about removing or rewording items. Finally, the provisional items were modified based on consensus in the project group. This resulted in a modified list of proposed items.

#### B) Interviews with a purposeful sample of people with and without (chronic) health conditions

The modified item list was tested by conducting interviews in a purposeful sample of people with and without (chronic) health conditions. The PROMIS® scientific standards specify the study sample should represent targeted sample of respondents of the concept of interest [25]. As our overall aim is to improve the measurement of participation of individuals independent of their (chronic) health conditions, we aimed to include adults with and without (chronic) health conditions. The inclusion criteria were being 18 years or older and able to read and have a conversation in Dutch. Eligible adults were recruited via a physician assistant working at the Department of Rehabilitation Medicine at Amsterdam UMC, location AMC in Amsterdam, and via the Dimence group (an organization offering mental welfare, well-being and social services) in Deventer, The Netherlands with the aim to include adults with physical conditions, mental conditions and their accompanying spouses/caretakers without a health condition. At location AMC, the physician assistant of the Department of Rehabilitation Medicine explained the study in short when an eligible adult visited the Department. At Dimence group, a practitioner recruited eligible adults. When an individual indicated to be willing to participate, MR called him/her and explained the study in more detail and scheduled the study interview in case someone was indeed able and willing to participate. Recruitment of new individuals ceased when, in line with the PROMIS® guidelines, the sample constituted of at least one person of ethnic minority, at least one with limited reading literacy, and at least one with a cognitive impairment [26].

Participants were interviewed by a researcher trained in qualitative research methods (MR) at Amsterdam AMC or at Dimence group in Deventer. Prior to the interview, written informed consent was obtained. The item list was presented on paper and interviews were held in Dutch, based on the think-aloud method [30]. Participants were asked to provide verbal open-ended feedback on whether the items were relevant and comprehensible, and if they had suggestions for improvement [27]. Participants were encouraged to verbalize their thoughts while reading the item. In addition, participants were invited to share any additional comments about the item list. All interviews were audio-recorded and transcribed verbatim. Qualitative content analysis was applied. Comments and problems were labeled based on content (‘meaningful concepts’) and subsequently grouped into categories by MR and LvL individually. Discrepancies between both researchers were resolved via discussion. All comments were taken into consideration. This resulted in a second modified list of proposed items.

### Step 3: item revision based on consensus in the project group

Items were selected and modified based on consensus in the project group. Per subdomain, one item was selected for inclusion in the final list of proposed items. Whether or not to preserve items was based on the results of the interviews with participants and the experience of and discussion by the project group members.

## Results

### Step 1: item generation

The item generation process resulted in a list of 48 proposed items. The items are shown in Supplemental Material 1. For example, for the subdomain ‘acquiring a place to live’ (see first row, Table 1), three items were drafted: (1) “I have trouble doing all the activities that are needed to acquire a place to live”, (2) “I have trouble doing everything needed to acquire a place to live”, and (3) “I feel limited in my ability to acquire a place to live”.

### Step 2: content validity

#### A) Content expert review

The 48-item list was discussed by 20 stakeholders during the expert meeting. The results from this step are presented per content validity aspect below. Examples and/or quotes of the issues are presented in Supplemental Material 2.

##### *Relevance*

With regard to the ratings on the relevance of the individual items, experts rated all items as relevant (> 50%). However, issues were reported during the group discussion:

1. Stem formulation and the PROMIS® definition of social participation
  The different formulations per subdomain caused confusion. It was discussed what stem formulation would be most relevant for the generated subdomains in relation to the construct of social participation.
2. Items measuring motivation instead of the effect of health on participation
  Some experts argued that some of the items did not seem to measure ‘purely’ the ability to participate in social roles and activities. Specifically, it was indicated that the answer of some items could be influenced by someone’s ‘motivation to participate’ rather than actual ability to participate in social roles and activities.
3. Calculating subdomain scores of social participation
  It was discussed whether it might be useful in the future to be able to calculate scores for subdomains. With regard to participation in work, it was mentioned that it might be helpful if there was a short form that contains specific questions that could be used in research specifically focussed on paid working populations.

##### *Comprehensibility*

With regard to comprehensibility of the items, the experts discussed several improvements:

1. Items’ interpretability being time-dependent while the original items are not
  With regard to items with the formulation ‘I feel’ in it, it was mentioned that answering this item could be time-dependent. Therefore, it was considered that items with ‘I feel’ could easily be answered differently depending on the time, day, and place. This may be problematic as it is expected that the degree of participation itself does not fluctuate during the day, hampering the reliability of these items.
2. Difficulty in applying the PROMIS® formulation to the formulation of proposed items
  It was discussed to what extent the standard stem formulation of PROMIS® was required. It was noticed that, for some items, more simple sentences could be used.
3. Textually vague formulations
  Items that contained formulations such as ‘I have trouble doing all activities needed for [..]’, or ‘I have trouble doing everything for [..]’, were reported as too vague. It was unclear what was meant with ‘all’ and ‘everything’.

##### *Comprehensiveness*

Content experts identified one additional subdomain: the use of social media. They viewed participation in social media as a main aspect of maintaining social relations with others for many people in today’s society, and therefore considered this subdomain an important addition to the proposed item list.

In the last part of the group discussion, content experts discussed the addition of items deemed relevant for people with high levels of participation. The following subdomain was identified: ‘participating in various social roles simultaneously, i.e., being a partner, a parent, an employee, a friend, et cetera, at the same time. The experts agreed that this is an ultimate participation item, because finding a good balance between these different social roles is challenging, also without disability or with a good health status.

#### B) Item modification

LvL, ST, MR, and CT critically reviewed and discussed all comments on the proposed item list. With regard to the definition of the construct of social participation, it was agreed that the construct should be defined as whether people have the capacity to do the activity. Although problems were identified with some of the item formulations, it was decided to retain multiple item formulations in the item list, to test them in step 2B. Where possible, items were simplified. For example, the item ‘I feel limited in the amount of time to get all the stuff and services needed for my daily life’ was reformulated into ‘I feel limited in my ability to go shopping’. In addition, items containing ‘all’ and ‘everything’ (see aforementioned results) were rewritten by removing these words from the item. For the additional subdomains about participating in social media and combining multiple social roles, which were identified during the group discussion, we again adhered to the formulation of the items included in the original item bank (same manner as described in the Methods section, Step 1B). Based on these two new subdomains, 9 more items were proposed. In total, the number of proposed items was 57. The items are shown in Supplemental Material 3.

#### C) Interviews with a purposeful sample of people with and without (chronic) health conditions

Ten adults participated in the interviews. Table 4 shows their characteristics. With regard to comprehensibility of the items, six items were considered not comprehensible by more than two participants; including one item on assisting others, one item on formal relationships, one item on non-remunerative employment, two items on economic transactions, and one item on community life. Participants’ suggestions for improvement were categorized in five categories. Examples and/or quotes of the issues are presented in Supplemental Material 4.

**Table 4:** Characteristics of participants involved in interviews (*n* = 10)

Variable	
Gender: male/female	5/5
Disability type, *n* Somatic (diabetes)	5
Psychological (autism)	2
No disability	3
Cognitive impairment	1^a^
Ethnic minority	1^a^

^a^Numbers do not add up as some participants fell into several categories

1. Formulation with ambiguous words [comprehensibility]
  Three participants indicated problems with items containing the formulation ‘I am limited’/‘I feel limited’. They had difficulty understanding what the limiting factor should be. In addition, with regard to the formulation ‘I am limited in my ability to..’, two participants considered it unclear what ‘ability’ meant. The Dutch word for ability in this item formulation can have two interpretations: ‘ability’ and ‘wealth’.
2. Formulations with multiple difficult words [comprehensibility]
  Some participants reported that the items containing multiple difficult words were too complex. For example, problems were encountered with the item ‘I feel limited in the amount of time I have to take care of my relatives and animals’. Moreover, it was observed that these items were too difficult to understand for the participant with a cognitive impairment.
3. In-depth questions
  Two participants expressed their preference for another way of item construction, starting with a more basic item and followed by an in-depth item when a problem was indicated. They mentioned that the in-depth items then should focus on which problems are experienced.
4. Relevance
  Two participants wondered whether participation in romantic relationships is relevant for measuring social participation. Two participants did not consider religion part of social participation. In addition, another participant suggested that for some items it should first be asked if the item was relevant to the person, and if so, the question about the subject could be asked. He referred to religion and volunteer work. Also, one participant wondered whether making digital economic transactions and using social media would be relevant for older people, but recognized its integral place in today’s society.
  Participants indicated that they found it confusing to answer multiple items on the same subject. However, which formulation was preferred differed. For example, one participant reported that the formulation about ability was the best, because of its practical nature, whereas another participant preferred the items with ‘I feel’, because of its subjective nature.
5. Comprehensiveness
  The additional item list was considered comprehensive and complete as no suggestions were made to include additional subdomains by the participants.

Data saturation results are presented in Supplemental Material 5.

### Step 3: item revision based on consensus in the project group

Based on the comments made in the interviews with the general population, discussion within the project group, and the concern about the feasibility of completing a long item list when testing the psychometric properties of the original item bank with the proposed items, it was decided to retain one item per subdomain and to remove the item on participating in religious and spiritual activities. As a general rule, it was agreed that the items with the stem formulation ‘I have trouble with’ were preferred due to their simple formulation. For some items, the stem formulation ‘I am limited’ was chosen by the research group when judged more appropriate for the meaning of the particular item/ subdomain. In total, 17 items were included in the final item list. The items are shown in Supplemental Material 6.

## Discussion

In the present study we aimed to generate items for subdomains of participation that are currently missing in the PROMIS® item bank v2.0 ‘Ability to Participate in Social Roles and Activities’ and evaluated their content validity. In total 17 items, covering 17 subdomains, were proposed to be added. Overall, the proposed items were perceived to be relevant and comprehensible, and the final item list was perceived to be comprehensive, as indicated by content experts and participants from a purposeful sample of people with and without (chronic) health conditions. The results therefore preliminarily support the addition of these items to the original item bank for further psychometric testing. With this qualitative study, a first step is taken towards an item bank that covers all (ICF) subdomains relevant to the general population, including items relevant for adults with a high level of participation. To our knowledge, we are the first to propose additional items to this item bank.

According to the content experts, participating in social media is an indication of an individual’s degree of social participation and more than just a way of interpersonal interaction, as the participation in social media is easy and passive and cannot always be replaced by other ways. This was supported by the participants as they found this subdomain relevant. Participation in social media was therefore added as an important subdomain of participation in social roles and activities. It has become an integral part of maintaining interpersonal relations [14]. As explained in the introduction, the added value of IRT-based item banks is that items, such as (technological) developments/ social innovations that are important for social participation, can be added, and items that become less relevant over time can be removed. One may question whether social media is a way of maintaining interpersonal relations rather than a subdomain of participation itself. Most people are nowadays very active with digital resources and the use of social media is becoming an essential part of our communication/ social connectivity. Further research should indicate whether participation in social media is indeed a relevant and valuable addition to the item bank.

### Methodological considerations

The distinction between functions, activities and participation is not always straightforward. They interact with each other, which is also depicted in World Health Organization’s ICF model [6]. This ‘issue’ was also experienced in the expert discussion with regard to the stem formulation of the items. To illustrate, doing an activity is different from participating in an activity (e.g., doing a sport or participate in a sports club). This distinction was therefore considered during the development of the items. This was also one of the reasons to adhere to the stem formulations used in the original PROMIS® item bank, with the aim that the proposed items measure the same underlying construct. Moreover, it is known that a fixed formulation facilitates participants to complete a questionnaire [31]. Additionally, the item formulations in the original Dutch item bank showed sufficient content validity [19, 20].

We have identified additional subdomains and proposed new items that, based on our previous qualitative study and this study [8], are considered part of the construct “participation in social roles and activities (or social participation)”. This may potentially improve the measurement of social participation in the Dutch general population. However, further testing should show whether the new items indeed improve the psychometric properties of the item bank and, thus, should be added to the item bank [9]. To illustrate, the quantitative study of Hahn et al. showed that while the items were designed to measure the same construct, the IRT test results were not consistent with model expectations [9]. In line with these results, not all of our new proposed items may fit the IRT model. It needs to be assessed whether the full set of items still measures one single construct, and IRT model fit and content validity should be balanced to make a decision on including new items in the item bank.

We have used the ICF as a framework to organize the subdomains of participation and as a reference to label and formulate newly generated items. In line with PROMIS®, the ICF concept of participation started from health and not from the concept of participation. The definition of participation in the ICF model is ‘involvement in a life situation’ [6] and the definition of participation in the PROMIS® item bank is ‘the perceived ability to perform one’s usual social roles and activities’ [9]. Previous research has found that the subcategories of the ICF appear to be more exhaustive than the PROMIS® item bank [24]. The PROMIS® concept of role participation does not completely align with the ICF concept of participation. Based on previous work of Bruijning et al. and Elsman et al. on the development of participation questionnaires using the ICF model as starting point [32, 33], we assume that maintaining romantic relationships, work and controlling finances are common-related social roles (being a partner and employee) and activities (working, controlling finances). Their questionnaires for (young) adults with vision impairment includes items on romantic relationships, managing finance/ allowance and work, and showed that these were unidimensional scales with sound psychometric properties [32, 33]. However, if an item (subdomain) was not considered relevant in the current study, the item was removed. This is illustrated by the study of De Wind et al., in which it appeared that religion and spirituality (ICF code d930) was considered a subdomain of participation [8]. However, based on the results of the current study there was insufficient evidence to propose an item on religion and spirituality to the PROMIS® item bank.

Another consideration is that although the ICF classification system provides a basis for identifying subdomains and levels of participation [34], the translation of the description of the ICF category into item formulations sometimes made the items too complex, indicated by both the content experts and the interviewed adults. Therefore, for some of the items simplified versions of ICF’s category descriptions had to be formulated. For example, the item ‘I have trouble taking care of household and personal objects including animals, plants, and furniture’ (item 6, Supplemental Material 1) was simplified into ‘I have trouble taking care of my household’ (item 3, Supplemental Material 6). It may be questioned whether people give the same interpretation to the simplified item, and therefore whether the specific ICF domain is being measured.

The proposal of adding only one item per subdomain to the original item list was considered most appropriate for further testing, mainly because of feasibility reasons. Adding multiple items per subdomain for further testing may have had the potential advantage that the item with the best fit to the IRT model would end up in the final revised item bank. Furthermore, an adequate balance across content of the extended item bank should be further investigated, to retain a representative group of items in each subdomains in the final version of the item bank. Such ‘content balancing’ is preferred especially in an item bank, i.e., that subdomains are represented proportionally, preferably also in the CAT version [35].

One of the strengths of PROMIS® item banks is the fact that they are applicable to everyone. Therefore, in the current item bank the question on participation in paid work is combined with a question on participation in unpaid work. However, the study by De Wind et al. found that paid work has a different meaning than unpaid work and should thus ideally be measured separately with the expense of not being relevant for everyone [8]. Furthermore, both content experts and participants suggested that it might be advantageous if future study participants could indicate which subdomain of participation they find most relevant. Based on IRT methods, it would be possible to create subdomain-specific short forms. These short forms could still be scored on the same metric as the complete item bank or any other short form from the same item bank. Alternatively, some item banks (e.g., PROMIS® Sexual Function and Satisfaction item banks) include screener questions to select relevant items [36]. With regard to social participation, both strategies should be further investigated in future work.

### Strengths and limitations

The strength of this study is that we used qualitative methods to evaluate content validity and received direct input from experts and adults sampled as potential users of the item bank.

Some limitations to our study should be considered. The group of participants in which the proposed items were evaluated consisted of only 10 participants, which is small according to the PROMIS® guidelines [25]. However, this does comply with the COSMIN guidelines [27]. We may not have reached complete data saturation. However, as shown in Supplemental Material 5, limited new information emerged in the last interviews, including comments on the stem formulation of the items. The comments on the stem formulation were irrelevant as we adhered to PROMIS® stem formulations and the remaining comments were insufficient to schedule additional interviews in our opinion. In addition, we did not balance the sample for age and gender. Moreover, half of the participants included in the interviews experienced disabilities resulting from diabetes. Unfortunately, and despite great effort, a participant with low literacy was not found. Translation of our results to people with low literacy may therefore be limited. Another limitation is that the present study was conducted among Dutch speaking individuals, who participate in Dutch society. The proposed items should therefore not be added directly to other language-versions of the item bank. Whether our results apply to other languages, cultures, or settings should be subject of further research.

### Implications/relevance

The present study provides new insights in the conceptualization and operationalization of the concept of participation in social roles and activities. Although the proposed item list cannot be added to the original item bank yet, we did show that the proposed items are considered relevant for measuring participation in social roles and activities by content experts and a sample of the targeted study populations.

### Future directions

The added value of the proposed items now needs to be further tested in a large-scale field study, including IRT analyses. It is recommended to investigate whether the items improve the psychometric properties of the current PROMIS® v2.0 item bank, in terms of validity, measurement precision, and number of items required in a CAT. When studying the psychometric properties of the proposed items special care should be taken to verify whether the items still measure the same single construct, for example by comparing the results of confirmatory factor analysis and local dependence of the item bank with and without the proposed new items.

## Conclusion

The process of item generation and content validity evaluation of the proposed items to the PROMIS® item bank v2.0 ‘Ability to Participate in Social Roles and Activities’ resulted in 17 items, representing 17 subdomains, that may contribute to the measurement of social participation.

## Electronic supplementary material

Below is the link to the electronic supplementary material.

Supplementary file 1 (DOCX 16 kb)

Supplementary file 2 (DOCX 14 kb)

Supplementary file 3 (DOCX 14 kb)

Supplementary file 4 (DOCX 15 kb)

Supplementary file 5 (DOCX 16 kb)

Supplementary file 6 (DOCX 13 kb)

## References

[1] Schuring M, Mackenbach J, Voorham T, Burdorf A (2011). The effect of re-employment on perceived health. Journal of Epidemiology and Community Health.

[2] Deeg DJ, Bath PA (2003). Self-rated health, gender, and mortality in older persons: Introduction to a special section. The Gerontologist.

[3] Douglas H, Georgiou A, Westbrook J (2017). Social participation as an indicator of successful aging: An overview of concepts and their associations with health. Australian Health Review.

[4] van der Holst M, Groot J, Steenbeek D, Pondaag W, Nelissen RG, Vliet Vlieland TP (2018). Participation restrictions among adolescents and adults with neonatal brachial plexus palsy: The patient perspective. Disability and Rehabilitation.

[5] Weinstein M, Goldman N, Hedley A, Yu-Hsuan L, Seeman T (2003). Social linkages to biological markers of health among the elderly. Journal of Biosocial Science.

[6] World Health Organization (WHO) (2001). International classification of functioning, disability and health: ICF.

[7] Powell JM, Rich TJ, Wise EK (2016). Effectiveness of occupation- and activity-based interventions to improve everyday activities and social participation for people with traumatic brain injury: A systematic review. The American Journal of Occupational Therapy.

[8] de Wind A, van der Beek AJ, Boezeman EJ, Swenneker R, Anema JR, de Boer A (2019). A qualitative study investigating the meaning of participation to improve the measurement of this construct. Quality of Life Research.

[9] Hahn EA, DeVellis RF, Bode RK, Garcia SF, Castel LD, Eisen SV (2010). Measuring social health in the patient-reported outcomes measurement information system (PROMIS): Item bank development and testing. Quality of Life Research.

[10] Eyssen IC, Steultjens MP, Dekker J, Terwee CB (2011). A systematic review of instruments assessing participation: Challenges in defining participation. Archives of Physical Medicine and Rehabilitation.

[11] Hahn EA, DeWalt DA, Bode RK, Garcia SF, DeVellis RF, Correia H (2014). New English and Spanish social health measures will facilitate evaluating health determinants. Health Psychology.

[12] Embretson SE, Reise SP (2013). Item response theory.

[13] Nunnally JC (1994). Psychometric theory 3E.

[14] Wright KB (2016). Communication in health-related online social support groups/communities: A review of research on predictors of participation, applications of social support theory, and health outcomes. Review of Communication Research.

[15] Castel LD, Williams KA, Bosworth HB, Eisen SV, Hahn EA, Irwin DE (2008). Content validity in the PROMIS social-health domain: A qualitative analysis of focus-group data. Quality of Life Research.

[16] Bartlett SJ, Orbai AM, Duncan T, DeLeon E, Ruffing V, Clegg-Smith K (2015). Reliability and validity of selected PROMIS measures in people with rheumatoid arthritis. PLoS ONE.

[17] Ahmad FS, Kallen MA, Schifferdecker KE, Carluzzo KL, Yount SE, Gelow JM (2019). Development and initial validation of the PROMIS(R)-Plus-HF profile measure. Circulation Heart failure.

[18] van der Meij E, Anema JR, Huirne JAF, Terwee CB (2018). Using PROMIS for measuring recovery after abdominal surgery: A pilot study. BMC Health Services Research.

[19] Terwee CB, Roorda LD, de Vet HC, Dekker J, Westhovens R, van Leeuwen J (2014). Dutch-Flemish translation of 17 item banks from the patient-reported outcomes measurement information system (PROMIS). Quality of Life Research.

[20] Terwee CB, Crins MHP, Boers M, de Vet HCW, Roorda LD (2019). Validation of two PROMIS item banks for measuring social participation in the Dutch general population. Quality of Life Research.

[21] Hammel J, Magasi S, Heinemann A, Whiteneck G, Bogner J, Rodriguez E (2008). What does participation mean? An insider perspective from people with disabilities. Disability and Rehabilitation.

[22] Chang FH, Coster WJ (2014). Conceptualizing the construct of participation in adults with disabilities. Archives of Physical Medicine and Rehabilitation.

[23] Norlander A, Iwarsson S, Jonsson AC, Lindgren A, Mansson Lexell E (2018). Living and ageing with stroke: An exploration of conditions influencing participation in social and leisure activities over 15 years. Brain Injury.

[24] Tucker CA, Cieza A, Riley AW, Stucki G, Lai JS, Bedirhan Ustun T (2014). Concept analysis of the patient reported outcomes measurement information system (PROMIS((R))) and the international classification of functioning, disability and health (ICF). Quality of Life Research.

[25] PROMIS. (2013). Instrument Development and Validation Scientific Standards version 2.0. Retrieved October 30, 2019, from, https://www.healthmeasures.net/images/PROMIS/PROMISStandards_Vers2.0_Final.pdf.

[26] DeWalt DA, Rothrock N, Yount S, Stone AA (2007). Evaluation of item candidates: The PROMIS qualitative item review. Medical Care.

[27] Terwee CB, Prinsen CAC, Chiarotto A, Westerman MJ, Patrick DL, Alonso J (2018). COSMIN methodology for evaluating the content validity of patient-reported outcome measures: A Delphi study. Quality of Life Research.

[28] Johnson, C., Aaronson, N., Blazeby, J.M., Bottomley, A.F.P., Koller, M., et al. (2011). *EORTC quality of life group: guidelines for developing questionnaires modules*. Brussels: EORTC. Retrieved February 4, 2020, from, https://www.eortc.org/app/uploads/sites/2/2018/02/guidelines_for_developing_questionnaire-_final.pdf.

[29] Corbin JM, Strauss A (1990). Grounded theory research: Procedures, canons, and evaluative criteria. Qualitative Sociology.

[30] Van Someren M, Barnard Y, Sandberg J (1994). The think aloud method: A practical approach to modelling cognitive processes.

[31] Aaronson, N., Choucair, A., Elliott, T., Greenhalgh, J., Halyard, M., Hess, R., et al. (2011). User’s guide to implementing patient-reported outcomes assessment in clinical practice. International Society for Quality Life Research. Retrieved October 30, 2019, from, https://pdfs.semanticscholar.org/9f86/43e764e9ef42dd9715312e0f91d631a530f7.pdf. 10.1007/s11136-011-0054-x 22048932

[32] Bruijning JE, van Rens GHMB, Knol D, van Nispen RMA (2013). Psychometric analyses to improve the Dutch ICF Activity Inventory. Optometry and Vision Science.

[33] Elsman EBM, van Rens GHMB, Nispen RMA (2018). Psychometric properties of a new intake questionnaire for visually impaired young adults: The participation and activity inventory for young adults (PAI-YA). PLoS ONE.

[34] Kostanjsek N (2011). Use of The International Classification of Functioning, Disability and Health (ICF) as a conceptual framework and common language for disability statistics and health information systems. BMC Public Health.

[35] Gershon RC (2005). Computer adaptive testing. Journal of Applied Measurement.

[36] Flynn KE, Lin L, Cyranowski JM, Reeve BB, Reese JB, Jeffery DD (2013). Development of the NIH PROMIS (R) sexual function and satisfaction measures in patients with cancer. The Journal of Sexual Medicine.

