# Supplementary material for: Proposal to extend the PROMIS® item bank v2.0 ‘Ability to Participate in Social Roles and Activities’: item generation and content validity
Source: Qual Life Res. 2020 Jun 2;29(10):2851–61. doi: 10.1007/s11136-020-02540-3 (PMC7561593; doi:10.1007/s11136-020-02540-3)
Supplement: Supplementary file 1 — (DOCX 16 kb) [file 11136_2020_2540_MOESM1_ESM.docx]

**Supplemental Material 1. Generated items based on identified subdomains by De Wind et al. (2019)[8]**

**Please note that items were directly translated for this Appendix from Dutch to English, and were not back-translated*

| **ICF domain** | **ICF subdomain (ICF code)** | **Generated items (n=48)** |
| --- | --- | --- |
| Domestic life | - Acquiring a place to live (d610) | 1. I have trouble doing all the activities that are needed to acquire a place to live 2. I have trouble doing everything needed to acquire a place to live 3. I feel limited in my ability to acquire a place to live |
|  | - Acquisition of goods and services (d620) | 1. I have trouble acquiring all goods and services required for daily living 2. I feel limited in the amount of time I have for acquiring all goods and services required for daily living. |
|  | - Caring for household objects (d650)* | 1. I have trouble taking care of household and personal objects including animals, plants, and furniture 2. I am limited in taking care of household and personal objects including animals, plants, and furniture 3. I feel limited in taking care of household and personal objects including animals, plants, and furniture 4. I feel limited in the amount of time I have for taking care of household and personal objects including animals, plants, and furniture 5. I have trouble keeping up with my household responsibilities |
|  | - Assisting others* |  |
| Interpersonal relationships | - Relating with strangers (d730) | 1. I have trouble engaging with strangers 2. I have trouble creating contacts with strangers |
|  | - Formal relationships (d740) | 1. I have trouble creating and maintaining formal relationships, such as with my employers, or (voluntary)organization 2. I feel limited in my ability to create and maintain formal relationships, such as with my employers, or (voluntary)organization |
|  | - Romantic relationships (d760) | 1. I have trouble with creating or maintaining close romantic relationships such as with lovers or sexual partners 2. I am limited in creating or maintaining close romantic relationships such as with lovers or sexual partners 3. I feel limited in my ability to create or maintain close romantic relationships such as with lovers or sexual partners |
| Major life areas | - Education life (d810-d839) | 1. I am limited in doing my education or training 2. I have trouble doing everything for my education/ training that I want to do 3. I have to do my education/training for shorter periods of time than usual 4. I have trouble doing all my education/training activities that are really important to me. 5. I have trouble doing all of the education/training I want to do 6. I have trouble keeping up with my education/training responsibilities. |
|  | - Remunerative employment (d850) | 1. I am limited in doing my paid work or internship 2. I have trouble doing everything for my paid work or internship that I want to do 3. I have to do my paid work or internship for shorter periods of time than usual 4. I have trouble doing all of the paid work/internship activities that are really important to me. 5. I have trouble doing all of the paid work/internship activities that I want to do` |
|  | - Non-remunerative employment (d855) | 1. I am limited in doing my unpaid work 2. I have trouble doing everything for my unpaid work that I want to do 3. I have to do my unpaid work for shorter periods of time than usual 4. I have trouble doing all of the unpaid work activities that are really important to me. 5. I have trouble doing all of the unpaid work activities that I want to do |
| Economic life | - Basic economic transactions (d860) | 1. I have trouble making payments (such as using money to purchase food) 2. I am limited in making payments (such as using money to purchase food) |
|  | - Complex economic transactions (d870) | 1. I have trouble controlling my finances (administer bank account) 2. I am limited in controlling my finances (administer bank account) 3. I have trouble taking care of my regular personal economic resources (such as benefits, capital) |
| Community life, social and civic life | - Community life (d910) | 1. I have trouble doing all of my regular community activities with others such as charitable organization, clubs, social organizations 2. I have to limit community activities with groups of people such as charitable organization, clubs, social organizations 3. I have trouble doing all of the community activities such as charitable organization, clubs, social organizations that I want to do 4. I have trouble participating in group activities |
|  | - Religion and spirituality (d930) | 1. I have trouble doing all of my regular religious activities with others 2. I have to limit religious activities with groups of people 3. I have trouble doing all of the religious activities with groups of people that I want to do |
|  | - Political life and citizenship (d950) | 1. I feel limited in the extent to which I can be socially and politically involved 2. I feel limited in the amount of time I have for engaging in social and political life |
|  | - Recreation and leisure (d920) | 1. I am limited to go on vacation |
