# Supplementary material for: Proposal to extend the PROMIS® item bank v2.0 ‘Ability to Participate in Social Roles and Activities’: item generation and content validity
Source: Qual Life Res. 2020 Jun 2;29(10):2851–61. doi: 10.1007/s11136-020-02540-3 (PMC7561593; doi:10.1007/s11136-020-02540-3)
Supplement: Supplementary file 2 — (DOCX 14 kb) [file 11136_2020_2540_MOESM2_ESM.docx]

**Supplemental Material 2. Quotes/ Examples Experts**

| **Issues** | ***Examples and/or illustrative quote(s)*** |
| --- | --- |
| **Relevance** |  |
| (1) Stem formulation and the PROMIS® definition of social participation. | *“Is it whether people do it, or whether they have the capacity to do it”.* |
| (2) Items measuring motivation instead of the effect of health on participation | An example of this problem was encountered with the item ‘I have difficulty taking responsibility for my job’. Experts reported that “*everyone probably encounters these difficulties from time to time*”, but that this “*does not mean that you are not able to participate*”. |
| **Comprehensibility** |  |
| (1) Items’ interpretability being time-dependent while the original items are not | “*One does not feel the same throughout the day: you can feel differently at different times of the day*”. |
| (2) Difficulty in applying the PROMIS® formulation to the formulation of proposed items | - With regard to interpersonal relations, it was mentioned that item formulations such as *“I do not see my friends enough”* is clearer than ‘I have trouble with maintaining my relationships [..]’. - One expert wondered if another stem ‘I can (not)’ could be a possibility in the formulation of the items. |
