# Supplementary material for: Proposal to extend the PROMIS® item bank v2.0 ‘Ability to Participate in Social Roles and Activities’: item generation and content validity
Source: Qual Life Res. 2020 Jun 2;29(10):2851–61. doi: 10.1007/s11136-020-02540-3 (PMC7561593; doi:10.1007/s11136-020-02540-3)
Supplement: Supplementary file 3 — (DOCX 14 kb) [file 11136_2020_2540_MOESM3_ESM.docx]

**Supplemental Material 3. Modified item list based on expert review**

**Please note that items were directly translated for this Appendix from Dutch to English, and were not back-translated*

| 1 | I feel limited in my ability to acquire a place to live |
| --- | --- |
| 2 | I have trouble doing what is needed to acquire a place to live |
| 3 | I have trouble acquiring my groceries |
| 4 | I feel limited in my ability to acquire my groceries |
| 5 | I feel limited in the amount of time I have for taking care of my household |
| 6 | I feel limited in my ability to take care of my household |
| 7 | I have trouble taking care of my household |
| 8 | I feel limited in taking care of my loved ones, including animals |
| 9 | I have trouble taking care of my loved ones, including animals |
| 10 | I feel limited in taking care of my loved ones, including animals |
| 11 | I feel limited in the amount of time I have to take care of my loved ones, including animals |
| 12 | I have trouble engaging with strangers |
| 13 | I have trouble creating contacts with strangers |
| 14 | I have trouble creating and maintaining formal relationships, such as with my employers, or (voluntary)organization |
| 15 | I feel limited in my ability to create and maintain formal relationships, such as with my employers, or (voluntary)organization |
| 16 | I have trouble creating formal relationships at work |
| 17 | I have trouble maintaining formal relationships at work |
| 18 | I have trouble with creating or maintaining close romantic relationships |
| 19 | I am limited in creating or maintaining close romantic relationships |
| 20 | I feel limited in my ability to create or maintain close romantic relationships |
| 21 | I am limited in doing my education or training |
| 22 | I have trouble doing everything for my education/ training that I want to do |
| 23 | I have to do my education/training for shorter periods of time than usual |
| 24 | I have trouble doing all my education/training activities that are really important to me |
| 25 | I have trouble doing all of the education/training I want to do |
| 26 | I have trouble keeping up with my education/training responsibilities |
| 27 | I am limited in doing my paid work or internship |
| 28 | I have trouble doing everything for my paid work or internship that I want to do |
| 29 | I have to do my paid work or internship for shorter periods of time than usual |
| 30 | I have trouble doing all of the paid work or internship activities that are really important to me |
| 31 | I have trouble doing all of the paid work or internship activities that I want to do |
| 32 | I am limited in doing my unpaid work |
| 33 | I have trouble doing everything for my unpaid work that I want to do |
| 34 | I have to do my unpaid work for shorter periods of time than usual |
| 35 | I have trouble doing all of the unpaid work activities that are really important to me |
| 36 | I have trouble doing all of the unpaid work activities that I want to do |
| 37 | I have trouble arranging businesses online, such as making payments |
| 38 | I am limited in arranging online businesses, such as making payments |
| 39 | I have trouble controlling my finances (administer bank account) |
| 40 | I am limited in controlling my finances (administer bank account) |
| 41 | I have trouble with online banking |
| 42 | I have trouble doing community activities with others such as a social association or club |
| 43 | I have to limit community activities such as at a social association or club |
| 44 | I have trouble doing community activities such as a social association or club |
| 45 | I have trouble doing my religious/spiritual activities with others |
| 46 | I have to limit religious/spiritual activities with groups of people |
| 47 | I have trouble doing my religious/spiritual activities |
| 48 | I have to limit religious/spiritual activities |
| 49 | I feel limited in the extent to which I can be socially and politically involved |
| 50 | I feel limited in the amount of time I have for engaging in social and political life |
| 51 | I have trouble traveling, for example going on vacation or business trip |
| 52 | I have trouble using digital and social media, such as Whatsapp, email, Facebook |
| 53 | I have trouble performing all my social roles simultaneously, such as parenting, at work, and socially |
| 54 | I have trouble performing all my social roles that are important to me, such as parenting, at work, and socially |
| 55 | I feel limited in the amount of time I have for performing all my social roles, such as parenting, at work, and socially |
| 56 | I have trouble finding the right balance in performing my social roles, such as parenting, at work, and socially |
| 57 | I have trouble finding the right balance between what I need and what I want |
