# Supplementary material for: Proposal to extend the PROMIS® item bank v2.0 ‘Ability to Participate in Social Roles and Activities’: item generation and content validity
Source: Qual Life Res. 2020 Jun 2;29(10):2851–61. doi: 10.1007/s11136-020-02540-3 (PMC7561593; doi:10.1007/s11136-020-02540-3)
Supplement: Supplementary file 4 — (DOCX 15 kb) [file 11136_2020_2540_MOESM4_ESM.docx]

**Supplemental Material 4. Quotes/ Examples Participants**

| **Issues** | ***Illustrative quote(s)*** |
| --- | --- |
| (1) Formulation with ambiguous words [comprehensibility] | An example of this interpretation problem was encountered with the item ‘I am limited in my ability to obtain a house’. One participant said, *“If you have the ability, financially, you buy a house”* (participant 4, male). One participant indicated that the Dutch word for ‘participating in community activities’ is “*too difficult for many people to understand”* (participant 5, female) in the items 42-44 regarding participating in community activities (Supplemental Material 3). |
| (2) Formulations with multiple difficult words [comprehensibility] | *“I have to think four times about how I should answer this item. You are thinking about ‘feeling’, ‘limitations’ and ‘time’. What is meant here?”* (participant 1, male)*.* |
| (3) In depth-questions | Example: the item about which task difficulties are experienced could follow the item about difficulties in performing one’s work tasks. |
| (4) Relevance   - Romantic relationships - Economic transactions | - One participant mentioned that he found this “*too personal and private*” and the item “*did not say anything about her participation*” (participant 6, female). - “*These are increasingly becoming integral topics/elements in today’s society”* (participant 9, male). |
