# Supplementary material for: Proposal to extend the PROMIS® item bank v2.0 ‘Ability to Participate in Social Roles and Activities’: item generation and content validity
Source: Qual Life Res. 2020 Jun 2;29(10):2851–61. doi: 10.1007/s11136-020-02540-3 (PMC7561593; doi:10.1007/s11136-020-02540-3)
Supplement: Supplementary file 5 — (DOCX 16 kb) [file 11136_2020_2540_MOESM5_ESM.docx]

**Supplemental Material 5. Data saturation results**

After the first 8 interviews, the item list was amended based on the results and the last 2 interviews were held with the revised item list. In the last interviews, comments were also related to the stem formulation of the items. The comments on the stem formulation were irrelevant as we adhered to PROMIS® stem formulations.
