# Supplementary material for: Proposal to extend the PROMIS® item bank v2.0 ‘Ability to Participate in Social Roles and Activities’: item generation and content validity
Source: Qual Life Res. 2020 Jun 2;29(10):2851–61. doi: 10.1007/s11136-020-02540-3 (PMC7561593; doi:10.1007/s11136-020-02540-3)
Supplement: Supplementary file 6 — (DOCX 13 kb) [file 11136_2020_2540_MOESM6_ESM.docx]

**Supplemental Material 6. Final list of proposed items**

**Please note that items were directly translated for this Appendix from Dutch to English, and were not back-translated*

| 1 | I have trouble doing what is needed to acquire a place to live |
| --- | --- |
| 2 | I have trouble acquiring my groceries |
| 3 | I have trouble taking care of my household |
| 4 | I have trouble taking care of my loved ones, including animals |
| 5 | I have trouble engaging with strangers |
| 6 | I have trouble creating and maintaining formal relationships, such as with my employers, or (voluntary)organization |
| 7 | I have trouble creating and maintaining romantic relationships |
| 8 | I have trouble doing everything for my education/ training that I want to do |
| 9 | I am limited in doing my paid work or internship |
| 10 | I am limited in doing unpaid work |
| 11 | I have trouble arranging online business, such as making payments |
| 12 | I have trouble controlling my finances (administer bank account) |
| 13 | I have trouble doing community activities such as in a social association |
| 14 | I feel limited in the extent to which I can be socially and politically involved |
| 15 | I have trouble traveling, for example going on vacation or business trip |
| 16 | I have trouble using digital and social media, such as Whatsapp, email, Facebook |
| 17 | I have trouble dividing my time between my family, work, friends, leisure time and myself |
